# Evaluating the Consistency of Subjective Activity Assessments and Their Relation to Cognition in Older Adults

**DOI:** 10.3390/geriatrics6030074

**Published:** 2021-07-28

**Authors:** Cassandra R. Hatt, Christopher R. Brydges, Jacqueline A. Mogle, Martin J. Sliwinski, Allison A. M. Bielak

**Affiliations:** 1 Department of Human Development and Family Studies, Colorado State University, Fort Collins, CO 80523, USA; allison.bielak@colostate.edu; 2 NIH West Coast Metabolomics Center, UC Davis, Davis, CA 95616, USA; crbrydges@ucdavis.edu; 3 College of Nursing, Pennsylvania State University, State College, PA 16801, USA; jam935@psu.edu; 4 Prevention Research Center, Pennsylvania State University, State College, PA 16801, USA; mjs56@psu.edu

**Keywords:** leisure activity engagement, measurement of activity, cognitive performance, aging adults

## Abstract

(1) Background: Research examining whether activity engagement is related to cognitive functioning in older adults has been limited to using retrospective reports of activity which may be affected by biases. This study compared two measurements (estimated weekly versus reported daily), and whether these activity assessments were related to cognition in older adults; (2) Methods: Participants from US (*n* = 199) and Australian (*n* = 170) samples completed a weekly estimate of activity, followed by 7 consecutive days of daily reporting. Differences between weekly estimates and daily reports were found, such that estimations at the weekly level were lower than self-reported daily information. Multivariate multiple regression was used to determine whether total activity, activity domains and the discrepancy between assessment types (i.e., weekly/daily) predicted cognitive performance across three cognitive domains (fluid, verbal, memory); (3) Results: When activity assessments were totaled, neither predicted cognition; however, when activity was grouped by domain (cognitive, social, physical), different domains predicted different cognitive outcomes. Daily reported cognitive activity significantly predicted verbal performance (β = 1.63, *p* = 0.005), while weekly estimated social activity predicted memory performance (β = −1.81, *p* = 0.050). Further, while the magnitude of discrepancy in total activity did not significantly predict cognitive performance, domain specific differences did. Differences in physical activity reported across assessments predicted fluid performance (β = −1.16, *p* = 0.033); (4) Conclusions: The significant discrepancy between the measurement types shows that it is important to recognize potential biases in responding when conducting activity and cognition research.

## 1. Introduction

The “Use it or Lose it” hypothesis of cognitive aging [1], also known as the engagement hypothesis, proposes that more time spent engaging in intellectual, social, and physical activities results in more optimal cognitive outcomes, protects against age-related cognitive decline, and lowers the risk for dementia [2]. Practicing such engagement in leisure activities in older adulthood may therefore buffer the effects of age-related cognitive declines and help promote successful aging. While there is some empirical support for the relation between activity and cognition [3], the evidence is far from conclusive and a number of questions and concerns have yet to be answered surrounding the precise nature of this relationship [4,5]. One issue is the time scale over which activity participation is measured, as studies have shown that information reported over different timeframes relates differently to cognition [6]. Specifically, the present paper examined whether discrepancies existed between subjective activity reports that were based on two different reported time frames: weekly estimated versus daily reported activity. Further, given the focus on the cognitive benefits of activity participation, we evaluated how well activity assessments with different time frames predicted overall cognitive performance, and whether the magnitude of the discrepancies in activity between the two frames was related to cognition. 

Activity engagement tends to be evaluated using self-report questionnaires of the frequency of typical activity over a certain time frame such as the past week, month, or year (see [4] for a review). The assessment of self-reported activities generally involves the presentation of a list of activities, and individuals are asked to estimate the number of hours they are engaged in each. The challenge with estimating activity participation is that subjective estimations may not be very accurate. For example, research has documented discrepancies between objective and subjective accounts of physical activity participation [7,8]. In general, participants tended to report less sedentary time than what was recorded using objective accelerometer recordings [7]. Outside of the physical activity literature, whether these discrepancies also exist for other forms of leisure activity, such as those involving social and cognitive engagement, is unknown. Objective accounts of these types of activities cannot be obtained as with physical activity information, and because only subjective reports of activity are available, researchers must assume that the information being collected is accurate. However, it is unclear whether the time frame of such self-reported information results in differences in reported information.

Estimates of activity engagement are susceptible to various inaccuracies and biases, including interpretation of the questionnaire itself. For instance, individuals may by confused by questionnaire phrasing, misunderstand the scope of activities to include in answers, and/or misunderstand the time frame of activities they were supposed to report, leading to struggles in accurately estimating the frequency and duration of their activity engagement [9]. Salthouse, Berish, and Miles (2002) discovered that when participants were asked to estimate the duration of engagement in 22 mental and social activities over the course of a typical week, several individuals reported activity totals of more than 24 h a day, suggesting that estimated accounts of activity information may not represent realistic activity patterns [10].

Even more distant retrospective reports of leisure activity (e.g., over the past two years) may be subject to reduced accuracy in reporting given evidence that episodic memory declines with age [11]. Cognitive biases may influence self-reports or estimates of typical activity. Motivation and the emotional experience associated with specific activities likely also influence the relevance and availability of activity-based memories [12,13]. Further, since perceived meaningfulness of experience impacts participation in activities [14], it is conceivable that this could also impact the accuracy of reported activity information. Activities that older adults are highly motivated to engage in but do not exceed their perceived abilities may be more accurately reported compared to other less motivating activities that are perceived as more difficult or associated with feelings of incompetency [15,16]. Individual differences in the strategic processes employed for emotional attention and memory might further explain why older adults would be expected to have difficulty in accurately remembering non-emotional activity information, especially when estimating over longer periods of time [17]. 

In contrast to estimating activity participation over longer time intervals, recording activity daily may offer advantages. When activities are written down chronologically shortly after they are undertaken, the tendency to under- or over-report certain activities may be diminished [18]. Daily reports may also be less susceptible to threats of bias and less limited by issues related to retrospective self-reporting [4,5,19,20]. When an individual is asked to remember the activities they engaged in throughout the past day (e.g., 24-h period), this information is easier to retrieve because it was more recently encoded and fresher in their minds [21].

When less time has elapsed between actual participation and activity assessment, there is less opportunity to forget activity related information. This is especially true because different types of activities elicit different emotional responses and these responses can impact what an individual remembers about their day versus what they forgot in terms of the type and frequency of activity engagement [12,15]. We reason that longer recall times may contribute to a greater chance of incomplete, inaccurate, or distorted self-reported activity information. Thus, we adhere to the assumption that activities that are reported over a shorter time frame (i.e., at the daily level) may be less susceptible to error and provide a more realistic depiction of activity engagement. Analogous to this assumption, Almeida (2002) argued that recalling stressors at the daily level produces information that is less affected by memory distortions. As the amount of time between the actual event and requested recall increases, the accuracy of information pertaining to the stressful encounter is reduced [22]. 

The present study focused on contrasting self-reported activity engagement by older adults across two different assessment periods: estimated weekly versus reported daily. Researchers have relied heavily on the notion that retrospective reports of activity information are accurate and outside of the physical activity literature [7], the phenomenon of discrepancies in reported activity information has never been tested. Samples from two different studies, one conducted in the United States and the other conducted in Australia were used to test the phenomenon of discrepancies in self-reported activity information. Our first aim was to evaluate whether older adults’ estimates of typical weekly activity matched the total summation of daily reported activity over a 7-day period. Given the greater likelihood for biases and errors in recall for the activity estimates [10,23,24], we hypothesized that total time spent in activity based on weekly estimates would be significantly higher than the totals of daily activity reports. Second, we examined whether one activity assessment (daily or weekly) was a stronger predictor of cognitive performance. In line with our prior reasoning that a shorter assessment window may provide a less biased report of activity [12,19], we expected daily reports to more strongly correspond to cognitive performance in older adults. 

Finally, we were interested in whether cognitive functioning differed for individuals whose weekly and daily assessments were relatively similar, and those who had large discrepancies between their weekly and daily activity totals. Given the aforementioned cognitive biases, and the roles of motivation, attention, and emotional attachment on the ability to accurately recall activity related information [14], individual differences in the ability to accurately recall activity related information may predict cognitive performance. We hypothesized that individuals who have smaller discrepancies between the two activity assessments may be more capable of providing consistent information; these individuals likely have better cognition than others who show greater mismatch between weekly estimates and daily reports of activity. That is, individuals with greater discrepancy between the two activity assessments may have poorer cognitive functioning compared to those individuals who reported more consistently across the two assessment periods. 

## 2. Materials and Methods

Data from two samples, the Activity Characteristics and Cognition (ACC) study [6] and the TRAnsitions In Later Life (TRAILLs) study [25] were used to assess our research questions. Both samples were used to assess the first question investigating differences in self-reported leisure activity information based on the assessment time frame. However, the second and third aims evaluating the link with cognitive performance were only conducted with the ACC sample. 

### 2.1. Participants

#### 2.1.1. ACC Study

Data from 207 community-dwelling adults aged 60–90 years old living in the Fort Collins, Colorado, and surrounding areas was collected. Recruitment began in 2012 and was targeted towards local aging community organizations through flyers, emails, and advertising in newsletters. The following inclusion criteria were applied: They (a) had to be at least 60 years of age, (b) willing to participate in the entire study, (c) have English as their primary language, (d) have no diagnosis of Alzheimer’s disease or other forms of dementia, psychotic disorder or personality disorder, (e) could see and hear clearly with corrective aids, and (f) were not full-time caregivers. All participants scored a 26 or higher on the Mini-Mental State Examination [26]. Eight participants were removed from the analyses for missing data (as described below). The remaining *n* = 199 participants (*M*_age_ = 70.74; *SD =* 6.79, 60 men, 139 women) were relatively healthy, rated themselves as having excellent or very good health (70.1%), and were highly educated (*M* = 16.40 years; *SD* = 2.54). Participants had the opportunity to earn up to $60 for completing the study, commensurate with their participation in the various parts of the study. There was limited attrition over the course of this study; only two participants were excluded from the analyses for not answering any days of the activity questionnaire and six individuals were removed for missing five or six days. 

#### 2.1.2. TRAILLs Study

This sample consisted of data from 185 Australian adults aged 51–84 years. Participants agreed to participate in the study by accepting an e-mail invitation link that was sent to all members of a local nonprofit organization (National Seniors Australia) in 2010. Of the original sample, fifteen participants were excluded from the present analyses for not completing at least one of the daily activity questionnaires. The remaining *n =* 170 participants (*M_age_* = 61.98, *SD* = 6.17) comprised the sample used for the present analyses (72 men, 93 women). These individuals were highly educated (*M =* 15.40 years; *SD =* 4.00), and relatively healthy (68.7% rated themselves as in excellent or very good health). Participants were not compensated for their participation in this study. 

### 2.2. Procedure

For both studies, participants completed an estimate of typical weekly activity first, followed by a week of daily reports to recreate two actual activity measurement methods used in studies. This is in contrast to having participants complete a daily report for a week, and then recall their weekly total activity for that same week. This second method would be influenced by daily reminders from the daily reports and would not represent how activity estimates are typically collected in the literature.

#### 2.2.1. ACC Study

Following providing consent for participation, participants completed a baseline assessment that included health, social, cognitive, and activity questionnaires. Baseline sessions typically took two hours to complete and were conducted one-on-one with trained research staff. A typical weekly estimate of activity was completed at baseline. Participants then completed identical activity questions to the weekly estimates every day for the next week (Week 1). Most participants completed the daily activity reports online (69.9%), while others with unreliable access to a computer were provided with a paper version of the activity reports as an alternative. During Week 2, participants completed a different open-ended activity daily diary (DD) (DD data not reported in the present analyses because the open-ended questions do not easily match the weekly estimate and daily report activities). Half of the sample completed the daily activity reporting as described (i.e., Week 1: daily activity reports; Week 2: DD), but half of the sample completed the open-ended DD in Week 1, followed by the daily reports in their second week. The online version of the daily report activities allowed access only once every 24 h. Individuals who completed the paper version of the assessment completed an honor pledge on days 3 and 6 confirming that they had in fact completed the questionnaire on the appropriate day and were instructed to leave any missed days blank. Participants received a phone call or email on the first day of the daily activity reports recording period and a reminder halfway through the week. See [6] for further detail of the study procedures. 

#### 2.2.2. TRAILLs Study

Participants who accepted the email invitation were given instructions linking them to the online survey site. During the first login, participants completed baseline questionnaires containing demographic, psychological, and social measures as well a weekly activity assessment. The week after initial login, individuals were asked to login to the site and complete up to 7 days of daily questionnaires assessing activity engagement. The participants were asked to complete the daily questionnaire sometime after dinner at around the same time each day within 2 weeks of their initial baseline assessment. Participants were asked to try and complete 7 consecutive days of logging in over the assessment period. 

### 2.3. Measures

#### 2.3.1. Activity Questionnaires

##### ACC Study

Twelve activities from the Activity Characteristics Questionnaire [6] were presented both at the baseline assessment (estimated weekly) and every day for 7 days (reported daily). These 12 items were chosen for their ease of estimation over a typical week, and ease of recall over a day. In the weekly estimate participants were asked, “In a typical week how much time do you spend engaged in the following activities?”: (1) Actively watching TV or movies, (2) Reading, (3) Writing, (4) Interacting with close friends, (5) Interacting with people who are not close friends, (6) Meeting new people, (7) Actively listening to information, (8) Attending community events, (9) Going out shopping, (10) Light-intensity activity or exercise, (11) Medium-intensity activity or exercise, and (12) Vigorous-intensity activity or exercise. The following responses were available: no time at all; some time but less than 15 min; 15 to 30 min; 31 min to 1 h; 1 to 2 h; 2 to 4 h; 4 to 8 h; 8 to 12 h; and 12 or more hours. In the daily assessment, participants were instructed to estimate the amount of time they had spent during the previous day (i.e., from midnight to midnight) engaged in the same activities. The same 12 activities listed above were questioned. The responses available for the daily assessment were the same as for the weekly assessment. 

For both the weekly estimate and daily reports the same coding scheme was followed: all ordinal responses were recoded into mean hours to create a continuous ratio scale variable. If a participant reported spending no time in an activity this was recoded as 0 h; some time to 15 min was recoded as 0.25 h; 15 to 30 min was recorded as 0.50 h; 31 min to 1 h was recoded as 1 h; 1 to 2 h was recoded as 1.5 h; 2 to 4 h was recoded as 3 h; 4 to 8 h was recoded as 6 h; 8 to 12 h was recoded as 10 h; and more than 12 h was recoded as 12 h. For time frames that were less than 1-h (e.g., some time to 15 min; 15–30 min) responses were recoded as the highest time possible. For all higher time frame options (e.g., 1–2 h; 2–4 h) responses were recorded as the average of that time frame. 

For the weekly estimate, the total time across all 12 activity types, and time estimated for each individual activity was calculated. For the daily reports, the reported hours for each activity type were summed across all seven days to give both total time for each individual activity, and a combined weekly total of hours reportedly spent engaged in all 12 types of activities. The percentage of individuals who completed all seven daily questionnaires was high: 95% of participants completed a full week of daily questionnaires; 4% completed six days; and 1% completed five days.

##### TRAILLs Study

The activity assessment included items adapted from the National Study of Daily Experiences [22] and preliminary versions of the Activity Characteristics Questionnaire [6,25]. At baseline (weekly estimate), participants were asked to estimate the amount of time they spent in a typical week participating in 28 different activities. Only seven of these activities were chosen for the present analyses to provide a close comparison to the types of activities included in the ACC study. The items were: (1) Going out shopping, (2) Vigorous-intensity activity or exercise, (3) Medium-intensity activity or exercise, (4) Light physical activity (5) Watching television, movies, videos, (6) Actively writing, and (7) Actively reading. 

For the daily reports, participants were asked to specify the amount of time they had spent engaged in the same 28 different activities as the weekly estimate, but only over the past 24 h. The response scales for both the weekly estimate (i.e., in a typical week) and the daily report (i.e., in the past 24 h) were the same and the following answer options were available: no time at all; between 0 and 15 min; 15 to 30 min; 31 min to 1 h; 1 to 2 h; 2 to 4 h; 4 to 8 h; 8 to 12 h; and 12 or more hours. The same recoding scheme was used for both the weekly estimate and daily report as was used for the ACC study. The weekly estimate total across all seven activity types, and for each individual activity was calculated. For the daily reports, the reported hours for each activity type were summed across all seven days to give a weekly total of hours reportedly spent engaged in each activity, and then combined across all seven types of activities. On average, participants completed 5.4 daily questionnaires, with a wide range in completion (25.3% of the participants completed all seven daily questionnaires, 32.4% completed six days, 17.1% completed five days, 12.4% completed four days, and 12.9% completed three days). 

#### 2.3.2. Cognitive Tests

##### ACC Study

All cognitive testing took place at the baseline testing session. Cognitive measures included: Digit Span (DS) Backward [27], a measure of working memory; the Trail Making Test (TMT) [28], where Part A measures processing speed and Part B taps into executive functioning; the Symbol Digit Modalities Test (SDMT) [29], a measure of processing speed; and Letter sets [30], a measure of reasoning ability. These four cognitive measures formed the Cognitive Fluid factor. The Cognitive Verbal factor included Controlled word associations [30] which assesses verbal fluency, and Vocabulary [30], a measure of verbal knowledge. Additionally, participants completed the Rey Auditory Verbal Learning Test (RAVLT) [31], which measures verbal learning (RAVLT-total trials 1–5), episodic memory (RAVLT-recall List A), and episodic memory-recognition (RAVLT Recognition). These three RAVLT scores formed the Cognitive Memory factor. Factor analysis of these cognitive measures has been documented elsewhere [6]. For each factor, scores on the individual cognitive tests were converted into *T* scores to allow for comparisons across measures and summed to create the total cognitive factor score (i.e., Fluid, Verbal, Memory). These procedures were specific to the ACC study, as comparable cognitive data from the TRAILLs study was not collected. 

### 2.4. Data Preparation and Statistical Analysis 

All data preparation and statistical analyses were performed using SPSS Statistics for Mac version 23. To address missing data in the ACC sample, mean imputation was used. Participants who recorded missing one or two days (*n* = 23) were given their individual mean for each question for the missing day, and those who missed individual questions on certain days also received their individual mean scores. In the TRAILLs sample, there was considerably more missing data, and 73 participants reported missing one or two days. As such we imputed data using the expectation maximization procedure in SPSS. Little’s (1988) Missing Completely At Random Test was nonsignificant, χ^2^(15) = 14.17, *p* = 0.51 meaning that the data was missing at random. 

First, to check that the two measures (weekly estimates and daily reports totaled across the week) in each sample were related, bivariate Pearson’s correlations were conducted. Large, significant correlations would imply that the two activity assessments are very closely related to each other, whereas moderate correlations between the two measures would provide support for our argument that the two assessments are related but potentially provide two different types of respondent information, based on the cognitive interferences previously discussed. Next, paired sample *t*-tests were used to evaluate differences between weekly estimated activity and reported daily activity (summed across 7-days) for both the ACC and TRAILLs data. We analyzed both differences in individual activity questions (across all activity types) and overall weekly activity (summed total across all activity questions). Differences were considered significant at the *p* = 0.01 level. We chose this more conservative significance cut-off to correct for multiple comparisons (i.e., *t*-tests), but adhered to the 0.05 cutoff for the other analyses described below as the multivariate nature of multivariate multiple regression (MMR) analysis inherently reduces the likelihood of type-1 error.

MMR was then conducted with the ACC data to determine whether either of the activity assessments (weekly or daily) predicted cognitive ability across the three cognitive factors: Fluid, Verbal, Memory. In addition to an MMR with total activity as the primary predictors, we conducted an MMR examining whether different activity domains for each assessment type predicted cognition. Cognitive (reading; writing; listening to new information), social (interacting with close and not close others; meeting new people; community events; shopping), and physical (light, medium, and vigorous intensity exercise) activity domains were created, informed by exploratory factor analyses and theoretical groupings consistent with other literature [6]. The activity of TV and movie watching was excluded from the total activity calculation and the activity domains as this activity has been consistently reported to have a negative association with cognitive ability. In this domain-specific MMR analysis, the three activity domains for the weekly assessment, three activity domains for the daily assessment, and covariates served as predictors of performance across three cognitive outcomes [6].

Finally, a separate MMR analysis was also conducted to determine if the amount of discrepancy between activity assessments (mean difference between weekly and daily activity) was related to performance across the three cognitive domains. In this analysis, difference scores for total activity reported across both weekly and daily assessments was calculated by subtracting the total reported daily activity (across all 7 days) from weekly estimated activity for each participant. An additional MMR analysis examined discrepancy by activity domain, where the difference scores for each of the three activity domains were simultaneously entered into the model predicting cognition.

## 3. Results

### 3.1. Differences between Estimated Weekly Activity and Daily Reported Activity

To contrast whether there were differences in the amount of activity hours reported using two different types of activity assessments (weekly versus daily) we conducted paired samples *t*-tests. Table 1 (ACC) and Table 2 (TRAILLs) provides descriptive statistics for all activities included in both studies. 

#### 3.1.1. ACC

The correlation between estimated weekly activity and total daily reported activity, across all 12 activity types, was small-to-moderate and positive, *r* (199) = 0.23, *p* = 0.001. A paired samples *t*-test found a significant difference between the total estimated weekly activity (hours/week) and total daily reported activity summed across the week (hours/week), *t* (198) = −34.52, *p* < 0.001, *d* = −3.05. Estimated weekly activity, collapsed across all 12 activity types, (*M* = 60.20, *SD* = 19.91) was lower than the total summation of daily reports across the week (*M* = 116.41, *SD* = 16.80). Table 1 shows that this difference was also apparent across all 12 types of activities, such that there were significant differences between estimated weekly activity and reported daily activity (hours/week) for all 12 activity types (all *p*s < 0.001). For each of the 12 types of activities measured in the ACC study, estimated weekly participation in that activity was significantly lower than the total summation of daily reported participation across the week. Therefore, participants consistently and significantly reported greater time in activity via the daily reports than on the weekly estimates. 

#### 3.1.2. TRAILLs

The bivariate correlation between weekly estimated activity and daily reports was positive and moderate-to-large, *r* (170) = 0.44, *p* < 0.001. There was also a significant difference between estimated weekly activity and total reported daily activity (across all seven activity types summed over the week), *t* (169) = −13.17, *p* < 0.001, *d =* 1.14. Consistent with the results for the ACC study, total estimated weekly activity (hours/ week) across all seven activities (*M* = 28.62, *SD* = 11.57) was lower than reported summed daily activity over the week (*M* = 49.02, *SD* = 22.48). Table 2 demonstrates that the pattern of mean differences between estimated weekly and reported daily activity over the week was consistent across nearly all the seven activity types (all *p* < 0.001). The exception was light physical activity, *t* (169) = 2.30, *p* = 0.02, *d* = *0*.23, which showed participants’ weekly estimations (*M =* 3.88, *SD =* 3.66) were greater than their total reported daily activity (*M* = 2.97, *SD* = 4.34); however, this test was not statistically significant based on the cutoff of *p* = 0.01. 

### 3.2. Predictors of Cognitive Performance

Using the ACC data, MMR was performed to examine which activity assessment, weekly or daily, predicted cognitive performance across three dependent variable factors: Fluid, Verbal, and Memory. Total activity for both assessments were entered as independent predictors, and age and years of education were both entered as covariates. 

The overall multivariate *R^2^* effect was significant, *F* (12, 505.63) = 6.03, *p* < 0.01, *R*^2^ = 0.44, indicating that the combination of the predictor variables significantly predicted performance on the three cognitive outcomes. Additionally, the predictor variables significantly predicted each cognitive dependent variable, Fluid factor, *F* (4, 193) = 9.60, *p* < 0.01, *R*^2^ = 0.15, Verbal factor *F* (4, 193) = 7.11, *p* < 0.01, *R*^2^ = 0.11, Memory factor, *F* (4, 193) = 7.47, *p* < 0.01, *R*^2^ = 0.12. When examining the parameter estimates however, it appears that these effects were primarily driven by the covariates, age, and education. Neither type of activity assessment total (weekly or daily) significantly predicted cognitive performance, but age and education had significant effects (*β* coefficients, *p* < 0.05) for Fluid and Verbal cognitive factors; memory factor performance was only significantly predicted by age (see Table 3).

To examine whether activity domains (cognitive, social, physical) differentially predicted cognitive performance, an additional MMR was conducted. Six activity domains (cognitive weekly, social weekly, physical weekly, cognitive daily, social daily, physical daily), plus the covariates age and education served as the independent variables in the model, and the three cognitive factors (Fluid, Verbal, Memory) were entered as dependent outcome variables. Overall, the multivariate *R^2^* was statistically significant *F* (24, 542.96) = 4.13, *p* < 0.01, *R^2^* = 0.42, indicating that the predictors collectively predicted performance on the set of cognitive outcomes. The multiple *R^2^s* for each of the three dependent variables were also significant, Fluid *F* (8, 189) = 5.37, *p* < 0.01, *R*^2^ = 0.15; Verbal *F* (8, 189) = 5.30, *p* < 0.01, *R*^2^ = 0.15; Memory *F* (8, 189) = 4.80, *p* < 0.01, *R*^2^ = 0.13. Upon examining the parameter estimates, these effects were driven mostly by the covariates, age and education (see Table 4). For the Fluid factor, none of the activity domains across either assessment were significant predictors; however, both age and education predicted performance. For the Verbal factor, in addition to the significant effects of age and education, daily cognitive activity also significantly predicted performance (*β* = 1.63, *p* = 0.005). For the Memory factor, performance was significantly predicted by age and weekly social activity (*β* = −1.81, *p* = 0.050). Correlation tables between predictor and outcome variables can be found under Supplementary Materials (Table S1).

Next, to examine how differences in estimation reports between total weekly and daily activity predicted cognitive performance another MMR was conducted. The difference score, age, and education served as the independent variables in the model, and the three cognitive factors (Fluid, Verbal, Memory) were entered as dependent variables. The difference score was computed by subtracting the total daily from the total weekly activity (For consistency with prior analyses, we maintained excluding TV in the discrepancy analysis as well. When additional analysis using all 12 activity items, including Watching TV/Movies, was performed the results remained non-significant). All parameter estimates for the total difference score MMR are presented in Table 5. The overall multivariate *R*^2^ was statistically significant, *F* (9, 467.43) = 7.87, *p* < 0.01, *R*^2^ = 0.54, indicating that age, education, and the amount of difference between activity estimates predicted performance on the set of cognitive outcomes. The multiple *R*^2^*s* for each of the three dependent variables were also significant, Fluid *F* (3, 194) = 12.67, *p* < 0.01, *R*^2^ = 0.16; Verbal *F* (3, 194) = 8.81, *p* < 0.01, *R*^2^ = 0.12; Memory *F* (3, 194) = 9.89, *p* < 0.01, *R*^2^ = 0.13. However, examination of the parameter estimates revealed that these effects were driven by the covariates, age, and education, as the difference score did not uniquely predict any of the cognitive outcomes (*β’s* were non-significant, *p* > 0.05 see Table 5).

In the final MMR analysis, activity domain difference scores were entered simultaneously as predictors of cognitive performance. Overall, the multivariate *R^2^* was statistically significant *F* (15, 524.91) = 5.37, *p* < 0.01, *R^2^* = 0.54, indicating that the predictor variables (age, education, cognitive activity difference, social activity difference, physical activity difference) collectively predicted performance on the set of cognitive outcomes. The multiple *R^2^s* for each of the three dependent variables were also significant, Fluid *F* (5, 192) = 8.61, *p* < 0.01, *R*^2^ = 0.16; Verbal *F* (5, 192) = 5.32, *p* < 0.01, *R*^2^ = 0.10; Memory *F* (5, 192) = 6.53, *p* < 0.01, *R*^2^ = 0.12. Upon examining the parameter estimates, it seemed that the effects were driven mostly by the covariates, age, and education (see Table 6). However, for the Fluid factor there was also a significant predictive effect of physical activity difference scores (*β* = −1.6, *p* =.033). This negative effect indicated that as the amount of estimation error between weekly and daily assessments for physical activity increased, performance on the fluid factor decreased. Correlation tables between predictor and outcome variables used in this analysis can be found under Supplementary Materials (Table S2).

## 4. Discussion

The present study aimed to systematically test for differences between two types of self-reported activity assessments: one that asked for a weekly estimate of activity, and another that asked for daily reports of activity participation. We also investigated whether these activity assessments were predictive of cognitive performance. Our findings showed that differences do indeed exist between activity assessments, where individuals tended to estimate significantly less weekly activity participation compared to when they reported their participation at the end of each day. Importantly, this difference was demonstrated in two different samples of older adults, from different countries. Interestingly, when activity assessments were totaled, neither predicted cognition; however, when activity was grouped by domain (cognitive, social, physical), different domains significantly predicted different cognitive outcomes. Further, while the magnitude of discrepancy in total activity did not significantly predict cognitive performance, domain specific differences did. These differential trends support the notion that using multiple forms of activity assessment may be rewarding for future research. 

In our study, both samples displayed significant differences between activity information based on a weekly estimate versus daily reports. A similar pattern of findings emerged across both samples: participants consistently reported less activity at the estimated weekly level than what was reported daily, across almost all activities. The moderate correlations found between the two measures in each sample demonstrate that the two assessments are related but potentially measuring two different constructs. The results show that different types of self-reported activity measurements, conducted over various time intervals, differ greatly in the resulting data. If the amount of activity time being reported is not consistent across days and weeks, then it is challenging to determine which assessments are collecting the most accurate activity information and under what conditions different assessments might be preferred or necessary. There are several reasons why there may have been discrepancies between daily reporting and weekly estimates. This includes that as more time passes between the moment of engagement to when activity is being assessed, cognitive biases become more pronounced and errors become more frequent [14,32,33]. This agrees with other lines of research suggesting that over greater periods of time, motivation and emotional experiences attached to different activities may influence the availability of activity-based memories [23]. Further, it is possible that participants tended to estimate different levels of participation at the weekly level compared to their daily reports as a result of cognitive biases and different heuristics used to recall activity related information [12]. At the same time, an alternative explanation for differences between the activity assessments may be the repeated questioning of activity every day. By completing daily questioning for a week, participants may have increased their awareness of what activities they commonly participated in, leading to reporting a greater number of hours engaged in activity. Further, the order of testing was such that the weekly estimate always preceded the daily reports, so greater awareness of engagement may have also been primed after completing the weekly estimate assessment. Since both daily and weekly assessments were subjective, it is difficult to determine which serves as a better proxy of accurate activity engagement. However, the present research demonstrates that differences are likely depending on time frame of assessment. 

The present study also investigated how strongly each type of activity assessment predicted cognitive performance. Given the shorter length of time over which daily assessment occurred and the multiple cognitive, social, and emotional influences that may operate on recalled activity information [14,32,33], we predicted that daily assessments would more strongly predict cognitive performance. Interestingly, total activity for neither assessment type predicted cognition. However, when activity was grouped into cognitive, social, and physical domains, both assessment types predicted different cognitive outcomes. At the daily level, higher cognitive activity predicted higher verbal factor performance, whereas at the weekly level, higher social activity predicted poorer performance on the memory factor. These findings demonstrate that both assessment types have predictive ability for cognition, but the specifics of that relation may differ depending on the nature of the activity. Cross-sectionally, past studies have consistently reported that engagement in cognitively stimulating activities favorably affects cognition. A study by Ferreira et al. demonstrated that activities such as engaging in puzzles was related to better verbal reasoning and working memory [34]. Other cross-sectional studies have also supported the basic premise that increased participation in cognitive, physical, and social leisure activities is related to higher cognitive performance [35,36,37]. Longitudinally, Ghisletta and colleagues reported more gradual declines in cognitive ability (e.g., perceptual speed) for individuals with higher levels of participation in cognitively stimulating activities such as reading, playing chess, and completing crossword puzzles [38]. While the influence of daily cognitive activity on cognition is in line with what prior research has demonstrated, the negative association between weekly social activity and memory performance was unexpected. Research conducted by Lövden and colleagues reported significant longitudinal associations between participation in social activities (e.g., sport, hobbies, playing games, attending cultural events), and perceptual speed over a six-year period, suggesting a dynamic lead-lag association between activity and cognition [39]. One possible explanation for this inconsistent finding is the potential overlap of functional aspects across different activity domains. For example, attending a community event may involve multiple domains of activity, as it occurs in a social setting, may involve cognitive skill-acquisition (e.g., listening to new information), as well as physical mobility to drive, walk, or bike to the location where the event is being held. This example demonstrates the difficulty with subjective interpretation of activity assessments and emphasizes the need for more research that analyzes the structural associations between different types of activities.

The findings reported here support prior research by Bielak [6] that used the same ACC sample, highlighting different effects on cognition based on the type of activity assessment. Both activity time reported in daily questionnaires and an activity questionnaire that recorded the frequency of participation over the past 2 years predicted unique variability in the cognitive factors. It seems that the specific type of activity scale used, the length of assessment time, and how the questionnaire is administered may all contribute to differences in reported findings.

The total amount of discrepancy between the two activity assessments did not significantly predict cognition, showing that how consistently individuals reported activity time was not related to better cognitive performance. However, when activity domains were used, differences in the physical activity domain predicted fluid factor performance. Greater discrepancy between weekly and daily physical activity predicted poorer fluid performance. This finding supported our hypothesis that individuals with greater differences in estimated activity would have poorer performance; however, this result was specific to physical forms of activity. It is unclear why greater discrepancy in physically specific activities was related to cognitive performance, and particularly only to fluid performance. If the discrepancy in activity reporting was telling of how cognizant a person is of their activity engagement, we would have expected the relation to apply to all three cognitive factors, including memory and fluid ability. This finding speaks to the value of examining activity types individually, collectively, and across different domains.

It is possible that different types of assessments provide different depictions of activity engagement because they are influenced by different underlying constructs. Asking participants to recall typical weekly activity patterns could make them more likely to draw upon self-schemas of what kind of person they believe themselves to be. For example, if a person views themselves as being physically fit and as leading an active and healthy lifestyle, they may be more likely to report that the activities they engage in, at the weekly level, are in line with these perspectives. This is in contrast to asking people to recall their activity over shorter periods of time such as at the daily level, which could allow for individuals to report less biased information about their recent activities without heavily relying on schematics or predispositions of what they typically do and how they perceive themselves [23,40]. This argument is in line with the heuristics and biases approach where people judge the likelihood of an event by the ease for which instances can be recalled [41]. At the daily level, activity reporting would be more consistent with the experienced self, which is present in the moment and representative of what an individual has actually been doing. On the other hand, estimated weekly activity would be more representative of the remembered self, which is influenced by availability and representativeness of what constitutes a “typical” week of activity for that person. These explanations are essential to consider when distinguishing findings from studies that used activity assessments with different time frames.

It is important to acknowledge that while our results were consistent across the two samples, differences between the ACC and TRAILLs studies including compensation, retention, compliance, and how assessment information was collected may have had implications on our findings. However, any impact likely would have been minimal. Differences in compensation between the samples may have impacted participants’ motivation to complete all parts of the study; however, attrition rates for both studies were quite low. Methods for retention were also different across the two samples, where email or phone call reminders of the daily questionnaire were provided in the ACC study. In contrast, there were no reminders provided in TRAILLs. The rates of compliance were also very different across studies; while 95% of participants completed a full week of daily questionnaires in the ACC study, only 25.3% of participants in the TRAILLs study completed all seven days of questionnaires. We tested for individual differences (age, gender, education, and self-rated health) associated with compliance rates in each study, but there were no significant differences between individuals who completed all seven days and those who completed less than the full week for either study.

Another difference between studies was the mode (online/paper) for which activity assessments were collected. In the ACC study, all participants completed a paper version of the activity assessment, in person, at the baseline testing session, followed by online daily questionnaires for the majority of the sample (69.9%). Those who did not have reliable access to a computer were provided an alternate paper version of the daily assessment. Differences in the mode of administration (online/paper) was not statistically controlled for in the ACC study but prior statistical models have shown no influence on activity results [6]. In the TRAILLs study, all participants completed both baseline (weekly) and daily assessments online and no paper alternative was offered. Since participants were not given an option for alternative paper testing in TRAILLS, if they did not have access to a computer they could not participate, influencing who participated in the study.

Lastly, the number of activity items included on the questionnaires differed across studies. Twelve activity questions were assessed in ACC versus seven in TRAILLs, and there were slight differences in the phrasing of the questions, including that ACC requested information on activity participation from midnight to midnight of the previous day, versus the past 24 h in TRAILLs. Even with the differences in study design between the two samples, our main finding that daily reports were higher than weekly estimations of activity, was consistent across both samples. Further, the slight variations in protocol in fact demonstrate the reliability of the findings.

One limitation of our study is that both samples consisted of relatively healthy, highly educated individuals, limiting the generalizability of our findings. While samples differed in their geographic location (i.e., United States and Australia), future research is needed using larger, more diverse cohorts. In addition, the study-design was cross-sectional; longitudinal research is warranted to better understand how the relationship between engagement in specific activities relates to cognitive performance across time. Additionally, because we did not have an objective record of activity engagement for each participant, we were unable to conclude that either type of assessment (estimated weekly or reported daily) is a more accurate representation of actual activity patterns in older adults. The issue of needing to identify ideal methods for measuring activity engagement, has been noted by others [4,5]. It may also be reasonable to use a variety of activity assessments, since multiple questionnaires seem to provide different information about activity engagement and how it relates to cognition [6].

To fully understand the relative importance of daily activity participation and its impact on cognitive functioning, more refined measurement tools and shorter data collection periods are suggested. It is advantageous to assess activity engagement multiple times per day, sampling moments in respondent’s lives through experience sampling methods [42,43]. The development of ecological momentary assessment (EMA), which captures individuals’ momentary responses within their daily lives, has marked a notable advance in the measurement of adult aging across the lifespan, while avoiding the distortions that may affect delayed recall, reducing the accuracy of self-reported information [43,44]. In fact, the best approach may be longitudinal studies that collect daily activity and cognitive information using EMA approaches in addition to longer term-assessments, providing the opportunity to evaluate how the relationship between lifestyle activity engagement and cognition operates at both the daily level and over years. Future studies that incorporate EMA will provide valuable insight into the nature of how the temporal relationship between activity engagement and cognition operates, and under what conditions and contexts activities are most beneficial for improving the daily lives and functioning of older adults.

## 5. Conclusions

This study compared two types of activity assessments (estimated weekly and reported daily) to evaluate the consistency of the information, and to determine if one was a stronger predictor of cognitive performance. This study is innovative because prior research has had no other choice but to assume that different assessments produce similar and consistent information about activity engagement, which does not seem to be the case. A shorter assessment period (reported daily activity) resulted in significantly greater reported activity time than what was estimated at the weekly level. The significant discrepancy between the measurement types shows that it is important to recognize potential biases in responding when conducting activity and cognition research. We considered daily reports of activity to be less influenced by cognitive bias and forgetting; however, both daily reported and weekly estimated activity domains predicted cognition but differentially, suggesting that each assessment type captured slightly different information [24]. This study is informative for aging research because it provides clear evidence, from two separate samples, that not all types of activity assessments provide similar, and consistent information. It may be advantageous for future research to use multiple types of assessments where activity is measured over different time frames. Multiple contextual, cognitive, and social influences seem to operate on the consistency of self-reported activity information, and our findings that estimated weekly and daily assessments produced inconsistent self-reported information, that differentially impacted cognition, underscore a clear need for measurement improvement and clarification [4].

## Figures and Tables

**Table 1:** ACC Study: Estimated Weekly and Reported Daily Activity (summed over 1 week) for 12 Activity Items (Hours/Week).

Activity Items	Weekly Estimate *M* (*SD)*	Daily Report (Weekly Sum) *M* (*SD*)	Mean Difference *M* (*SD*)	*p*
Actively Watching TV/movies	7.76 (4.29)	11.52 (3.56)	−3.77 (4.32)	<0.001
Reading	9.69 (3.21)	12.88 (0.68)	−3.20 (3.17)	<0.001
Writing	4.97 (3.93)	9.96 (4.06)	−4.98 (4.74)	<0.001
Interacting with close friends	7.65 (4.08)	12.37 (2.29)	−4.72 (4.24)	<0.001
Interaction with not close friends	5.37 (3.99)	12.14 (2.20)	−6.77 (4.38)	<0.001
Meeting new people	1.29 (1.86)	5.77 (4.44)	−4.47 (4.46)	<0.001
Actively listening to information	5.89 (3.87)	12.08 (2.53)	−6.18 (4.15)	<0.001
Attending community events	2.66 (2.50)	6.17 (4.91)	−3.52 (4.89)	<0.001
Going out shopping	3.00 (2.43)	9.54 (3.76)	−6.54 (4.11)	<0.001
Light-intensity activity/exercise	5.79 (3.94)	11.49 (3.14)	−5.70 (4.46)	<0.001
Medium-intensity activity/exercise	3.96 (3.11)	8.68 (4.75)	−4.72 (4.77)	<0.001
Vigorous-intensity activity/exercise	2.14 (2.93)	3.80 (4.92)	−1.66 (5.07)	<0.001
Total activity	60.20 (19.91)	116.41 (16.80)	−56.21 (22.97)	<0.001

Note. *p* values from paired-samples *t*-tests (*df* = 198 in all cases).

**Table 2:** TRAILLs Study: Estimated Weekly and Reported Daily Activity (summed over 1 week) for 12 Activity Items (Hours/Week).

Activity Items	Weekly Estimate *M* (*SD*)	Daily Report (Weekly Sum) *M* (*SD*)	Mean Difference *M* (*SD*)	*p*
TV, Movies, Videos	7.42 (3.71)	15.68 (9.18)	−8.26 (7.82)	<0.001
Actively Reading	5.65 (3.51)	9.17 (6.98)	−3.52 (6.07)	<0.001
Actively Writing	4.54 (3.74)	6.73 (7.42)	−2.19 (6.63)	<0.001
Going out shopping	2.19 (1.81)	4.83 (5.36)	−2.64 (5.37)	<0.001
Light physical activity	3.88 (3.66)	2.97 (4.34)	0.92 (5.19)	0.023
Medium-intensity activity	3.02 (2.89)	5.85 (6.00)	−2.84 (5.83)	<0.001
Vigorous-intensity activity	1.92 (2.57)	3.79 (3.98)	−1.86 (4.27)	<0.001
Total activity	28.62 (11.57)	49.02 (22.48)	−20.40 (20.20)	<0.001

Note. *p* values from paired-samples *t*-tests (*df* = 169 in all cases).

**Table 3:** Multivariate Multiple Regression Standardized Coefficients for Total Activity, Age, and Education Predicting Cognitive Factors.

Dependent Variables		β	*p*
Fluid factor ^a^	Age	−1.19	<0.001
	Education	1.79	0.003
	Weekly Estimate	−0.00	0.392
	Daily Report	0.08	0.971
Verbal factor ^b^	Age	−0.31	0.020
	Education	1.47	<0.001
	Weekly Estimate	0.07	0.180
	Daily Report	0.03	0.649
Memory factor ^c^	Age	−1.09	<0.001
	Education	0.30	0.585
	Weekly Estimate	−0.03	0.673
	Daily Report	0.09	0.303

^a^ Fluid Factor (Digit Span, TMT Difference, DSST, Letter Sets) *R*^2^ = 0.149. ^b^ Verbal Factor (Controlled Word Association, Vocabulary) *R*^2^ = 0.110. ^c^ Memory Factor (RAVLT Total, Recognition, Recall) *R*^2^ = 0.116.

**Table 4:** Multivariate Multiple Regression Standardized Coefficients for Weekly and Daily Activity Domains, Age, and Education Predicting Cognitive Factors.

Dependent Variables		β	*p*
Fluid factor ^a^	Age	−1.18	0.000
	Education	1.76	0.004
	Weekly Estimate		
	Cognitive Activity	0.20	0.770
	Social Activity	0.97	0.330
	Physical Activity	−1.27	0.089
	Daily Report		
	Cognitive Activity	0.03	0.973
	Social Activity	−0.33	0.701
	Physical Activity	1.16	0.062
Verbal factor ^b^	Age	−0.29	0.028
	Education	1.33	0.000
	Weekly Estimate		
	Cognitive Activity	0.35	0.364
	Social Activity	−0.22	0.694
	Physical Activity	0.57	0.179
	Daily Report		
	Cognitive Activity	1.63	0.005
	Social Activity	−0.75	0.117
	Physical Activity	−0.02	0.946
Memory Factor ^c^	Age	−1.07	0.000
	Education Weekly Estimate	0.19	0.727
	Cognitive Activity	1.04	0.095
	Social Activity	−1.81	0.050
	Physical Activity	0.052	0.940
	Daily Report		
	Cognitive Activity	1.00	0.281
	Social Activity	0.69	0.379
	Physical Activity	−0.14	0.799

^a^ Fluid Factor (Digit Span, TMT Difference, DSST, Letter Sets). Daily *R*^2^ = 0.149; Weekly *R*^2^ = 0.151. ^b^ Verbal Factor (Controlled Word Association, Vocabulary). Daily *R*^2^ = 0.147; Weekly *R*^2^ = 0.149. ^c^ Memory Factor (RAVLT Total, Recognition, Recall). Daily *R*^2^ = 0.125; Weekly *R*^2^ = 0.133.

**Table 5:** Multivariate Multiple Regression Standardized Coefficients of Total Activity Difference Score, Age, and Education Predicting Cognitive Factors.

Dependent Variables		β	*p*
Fluid factor ^a^	Age	−1.22	<0.001
	Education	1.85	0.002
	Total Activity Difference Score	−0.04	0.601
Verbal factor ^b^	Age	−0.34	0.009
	Education	1.54	<0.001
	Total Activity Difference Score	0.03	0.494
Memory factor ^c^	Age	−1.11	<0.001
	Education	0.35	0.526
	Total Activity Difference Score	−0.06	0.369

^a^ Fluid Factor (Digit Span, TMT Difference, DSST, Letter Sets) *R*^2^ = 0.151. ^b^ Verbal Factor (Controlled Word Association, Vocabulary) *R*^2^ = 0.106. ^c^ Memory Factor (RAVLT Total, Recognition, Recall) *R*^2^ = 0.119.

**Table 6:** Multivariate Multiple Regression Standardized Coefficients for Activity Domain Difference Scores, Age, and Education Predicting Cognitive Factors.

Dependent Variables	Predictors	β	*p*
Fluid factor ^a^	Age	−1.19	0.000
	Education	1.77	0.000
	Cognitive Activity Difference	0.23	0.707
	Social Activity Difference	0.56	0.432
	Physical Activity Difference	−1.16	0.033
Verbal factor ^b^	Age	−0.35	0.008
	Education	1.55	0.000
	Cognitive Activity Difference	−0.13	0.719
	Social Activity Difference	0.30	0.478
	Physical Activity Difference	0.15	0.640
Memory Factor ^c^	Age	−1.09	0.000
	Education	0.35	0.520
	Cognitive Activity Difference	0.50	0.384
	Social Activity Difference	−1.2	0.059
	Physical Activity Difference	0.06	0.914

^a^ Fluid Factor (Digit Span, TMT Difference, DSST, Letter Sets). Daily *R*^2^ = 0.149; Weekly *R*^2^ = 0.162. ^b^ Verbal Factor (Controlled Word Association, Vocabulary). Daily *R*^2^ = 0.147; Weekly *R*^2^ = 0.099. ^c^ Memory Factor (RAVLT Total, Recognition, Recall). Daily *R*^2^ = 0.125; Weekly *R*^2^ = 0.123.

## Data Availability

Data available upon request from the corresponding author.

## Supplementary Materials

The following are available online at https://www.mdpi.com/article/10.3390/geriatrics6030074/s1, Table S1: Correlations between Daily and Weekly Activity Domain Assessment Scores, Table S2: Correlations between Total Activity & Domain Differences, and Cognitive Factors.

## References

[1] Hultsch D.F., Hertzog C., Small B.J., Dixon R.A. (1999). Use it or lose it: Engaged lifestyle as a buffer of cognitive decline in aging?. Psychol. Aging.

[2] Wang J.Y.J., Zhou D.H.D., Li J., Zhang M., Deng J., Tang M., Gao C., Lian Y., Chen M. (2006). Leisure activity and risk of cognitive impairment: The Chongqing aging study. Neurology.

[3] Hertzog C., Kramer A., Wilson R., Lindenberger U. (2009). Enrichment effects on adult cognitive development: Can the functional capacity of older adults be preserved and enhanced?. Psychol. Sci. Public Interest.

[4] Bielak A.A.M. (2010). How can we not ‘lose it’ if we still don’t understand how to ‘use it’? Unanswered questions about the influence of activity participation on cognitive performance in older age—A mini-review. Gerontology.

[5] Salthouse T.A. (2006). Mental exercise and mental aging: Evaluating the validity of the “use it or lose it” hypothesis. Perspect. Psychol. Sci..

[6] Bielak A.A.M. (2017). Different perspectives on measuring lifestyle engagement: A comparison of activity measures and their relation with cognitive performance in older adults. Aging Neuropsychol. Cogn..

[7] Matton L., Wijndaele K., Duvigneaud N., Duquet W., Philippaerts R., Thomis M., Lefevre J. (2007). Reliability and validity of the Flemish Physical Activity Computerized Questionnaire in adults. Res. Q. Exerc. Sport.

[8] Miller D.I., Taler V., Davidson P.S., Messier C. (2012). Measuring the impact of exercise on cognitive aging: Methodological issues. Neurobiol. Aging.

[9] Heesch K.C., van Uffelen J.G.Z., Hill R.L., Brown W.J. (2010). What do IPAQ questions mean to older adults? Lessons from cognitive interviews. Int. J. Behav. Nutr. Phys. Act..

[10] Salthouse T.A., Berish D.E., Miles J.D. (2002). The role of cognitive stimulation on the relations between age and cognitive functioning. Psychol. Aging.

[11] Craik F.I.M., Park D., Schwarz N. (2000). Age related changes in human memory. Cognitive Aging.

[12] Mather M., Carstensen L. (2005). Aging and motivated cognition: The positivity effect in attention and memory. Trends Cogn. Sci..

[13] Robinson M.D., Clore G.L. (2002). Belief and feeling: Evidence for an accessibility model of emotional self-report. Psychol. Bull..

[14] Parisi J.M. (2010). Engagement in adulthood: Perceptions and participation in daily activities. Act. Adapt. Aging.

[15] Cacioppo J.T., Petty R.E., Feinsteinm J.A., Jarvis W.B.G. (1996). Dispositional differences in cognitive motivation: The life and times of individuals varying in need for cognition. Psychol. Bull..

[16] Verplanken B. (1989). Involvement and need for cognition as moderators of beliefs—attitude—intention consistency. Br. J. Soc. Psychol..

[17] Forstmeier S., Maercker A. (2008). Motivational reserve: Lifetime motivational abilities contribute to cognitive and emotional health in old age. Psychol. Aging.

[18] Juster F., Stafford F. (1991). Work and Leisure: On the Reporting of Poll Results: Comment. Public Opin. Q..

[19] Ariel R., Price J., Hertzog C. (2015). Age-related associative memory deficits in value-based remembering: The contribution of agenda-based regulation and strategy use. Psychol. Aging.

[20] Dudukovic N.M., DuBrow S., Wagner A.D. (2009). Attention during memory retrieval enhances future remembering. Mem. Cogn..

[21] Delaney P.F., Verkoeijen P.P.J.L., Spirgel A., Ross B.H. (2010). Chapter 3—Spacing and Testing Effects: A Deeply Critical, Lengthy, and at Times Discursive Review of the Literature. Psychology of Learning and Motivation.

[22] Almeida D.M., Wethington E., Kessler R.C. (2002). The daily inventory of stressful events. Assessment.

[23] Parisi J.M., Stine-Morrow E.A.L., Noh S.R., Morrow D.G. (2009). Predispositional engagement, activity engagement, and cognition among older adults. Aging Neuropsychol. Cogn..

[24] Hess T.M., Growney C.M., O’Brien E.L., Neupert S.D., Sherwood A. (2018). The role of cognitive costs, attitudes about aging, and intrinsic motivation in predicting engagement in everyday activities. Psychol. Aging.

[25] Curtis R.G., Windsor T.D., Mogle J.A., Bielak A.A.M. (2017). There’s more than meets the eye: Complex associations of daily pain, physical symptoms, and self-efficacy with activity in middle and older adulthood. Gerontology.

[26] Folstein M.F., Folstein S.E., McHugh P.R. (1975). A practical method for grading the cognitive state of patients for the clinician. J. Psychiatr. Res..

[27] Wechsler D. (2008). Wechsler Adult Intelligence Scale.

[28] Reitan R.M. (1992). Trail Making Test.

[29] Smith A. (1982). A Symbol Digit Modalities Test: Manual.

[30] Ekstrom R.B.E.A. (1976). Manual for Kit of Factor-Referenced Cognitive Tests.

[31] Schmidt M. (1996). Rey Auditory Verbal Learning Test: A Handbook.

[32] Hess T.M., Smith B.T., Sharifian N. (2016). Aging and effort expenditure: The impact of subjective perceptions of task demands. Psychol. Aging.

[33] Szanton S.L., Walker R.K., Roberts L., Thorpe R.J., Wolff J., Agree E., Roth D.L., Gitlin L.N., Seplaki C. (2015). Older adults’ favorite activities are resoundingly active: Findings from the NHATS study. Geriatr. Nurs..

[34] Ferreira N., Owen A., Mohan A., Corbett A., Ballard C. (2015). Associations between cognitively stimulating leisure activities, cognitive function and age-related cognitive decline. Int. J. Geriatr. Psychiatry.

[35] Vance D.E., Wadley V.G., Ball K.K., Roenker D.L., Rizzo M. (2005). The effects of physical activity and sedentary behavior on cognitive health in older adults. J. Aging Phys. Act..

[36] Bugg J.M., DeLosh E.L., Clegg B.A. (2006). Physical activity moderates time-of-day differences in older adults’ working memory performance. Exp. Aging Res..

[37] Newson R.S., Kemps E.B. (2006). The Influence of Physical and Cognitive Activities on Simple and Complex Cognitive Tasks in Older Adults. Exp. Aging Res..

[38] Ghisletta P., Bickel J.-F., Lövdén M. (2006). Does activity engagement protect against cognitive decline in old age? Methodological and analytical considerations. J. Gerontol. Ser. B Psychol. Sci. Soc. Sci..

[39] Lövden M., Ghisletta P., Lindenberger U. (2005). Social participation attenuates decline in perceptual speed in old and very old age. Psychol. Aging.

[40] Jopp D., Hertzog C. (2007). Activities, self-referent memory beliefs, and cognitive performance: Evidence for direct and mediated relations. Psychol. Aging.

[41] Kahneman D., Slovic P., Tversky A. (1982). Judgment under Uncertainty: Heuristics and Biases.

[42] Timmers C., Maeghs A., Vestjens M., Bonnemayer C., Hamers H., Blokland A. (2014). Ambulant Cognitive Assessment Using a Smartphone. Appl. Neuropsychol. Adult.

[43] Spooner D.M., Pachana N.A. (2006). Ecological validity in neuropsychological assessment: A case for greater consideration in research with neurologically intact populations. Arch. Clin. Neuropsychol..

[44] Sliwinski M.J., Mogle J.A., Hyun J., Munoz E., Smyth J.M., Lipton R.B. (2016). Reliability and Validity of Ambulatory Cognitive Assessments. Assessment.

