# Supplementary material for: Evaluating the Consistency of Subjective Activity Assessments and Their Relation to Cognition in Older Adults
Source: Geriatrics (Basel). 2021 Jul 28;6(3):74. doi: 10.3390/geriatrics6030074 (PMC8395599; doi:10.3390/geriatrics6030074)
Supplement: Supplementary file 1 [file geriatrics-06-00074-s001.zip › geriatrics-1271922-supplementary.pdf]

Table S1. Correlations Between Daily and Weekly Activity Domain Assessment Scores.

	Cognitive Daily	Social Daily	Physical Daily	Cognitive Weekly	Social Weekly	Physical Weekly
Cognitive Daily	1	0.26**	0.18*	0.26**	−0.02	0.00
Social Daily	0.26**	1	−0.20**	0.04	0.21**	0.10
Physical Daily	0.18*	0.20**	1	−0.04	0.07	0.39**
Cognitive Weekly	0.26**	0.04	−0.04	1	0.45**	0.26**
Social Weekly	−0.02	0.21**	0.07	0.45**	1	0.38**
Physical Weekly	0.00	0.10	0.39**	0.26**	0.38**	1

* Correlation significant at the 0.01 level (2-tailed). ** Correlation significant at the 0.05 level (2-tailed).

Table S2. Correlations Between Total Activity & Domain Differences, and Cognitive Factors.

	Total Activity Difference	Cognitive Activity Difference	Social Activity Difference	Physical Activity Difference	Fluid Factor	Verbal Factor	Memory Factor
Total Activity Difference	1	0.75**	0.81**	0.68**	−0.02	0.05	−0.03
Cognitive Domain Difference	0.75**	1	0.44**	0.34**	0.05	0.03	0.06
Social Domain Difference	0.81**	0.44**	1	0.28**	0.04	0.06	−0.09
Physical Domain Difference	0.68**	0.34**	0.28**	1	−0.14	0.02	−0.00
Fluid Factor	−0.02	0.05	0.04	−0.14	1	0.33**	0.31**
Verbal Factor	0.05	0.03	0.06	0.02	0.33**	1	0.33**
Memory Factor	−0.03	0.06	−0.09	−0.00	0.31**	0.33**	1

* Correlation significant at the 0.01 level (2-tailed). ** Correlation significant at the 0.05 level (2-tailed).
